# Developmental origins of immune function: Maternal prenatal mood is associated with infant immune cell gene expression

**DOI:** 10.1016/j.bbi.2025.106230

**Published:** 2025-12-19

**Authors:** Gabrielle R. Rinne, Christine Dunkel Schetter, Susan Jackman, Steve W. Cole

**Affiliations:** a Department of Psychology, University of California, Los Angeles, CA, USA; b Department of Psychology, University of Southern California, Los Angeles, CA, USA; c Cedars-Sinai Medical Center. Los Angeles, CA, USA; d Cousins Center for Psychoneuroimmunology, Department of Psychiatry and Biobehavioral Sciences. University of California, Los Angeles, CA, USA

**Keywords:** Pregnancy, Depression, Anxiety, Gene expression, Inflammation, Infancy

## Abstract

The in-utero environment shapes offspring mental and physical health trajectories over the lifespan, likely through developmental adaptations to fetal biological systems. Offspring immune system development is a putative pathway through which the prenatal environment influences offspring health. The current study tested associations of maternal prenatal depressive and anxiety symptoms with infant pro-inflammatory and antiviral gene expression in a sample of 118 mother-infant pairs enrolled in a longitudinal study. Mothers reported on depressive and anxiety symptoms during interviews in early, mid, and late pregnancy. About one month after birth, trained research staff collected dried blood spots from infants during a heel stick procedure (*M* = 1.3 months, *SD* = 1.1 months). Infant dried blood spots were assayed for genome-wide transcriptional profiles using RNAseq. We evaluated associations of maternal prenatal depressive and anxiety symptoms with infant genome-wide transcriptional profiles and used bioinformatics analyses to identify upstream transcriptional pathways of differentially expressed genes. Higher maternal depressive symptom levels over the course of pregnancy were associated with upregulation of the pro-inflammatory NF-κB transcription control pathway and downregulation of the antiviral IRF control pathway in infants. In contrast, anxiety symptoms were associated with downregulation of the antiviral transcriptional control pathway in infants but were not associated with differences in the pro-inflammatory transcriptional control pathway. However, the association of anxiety symptoms with antiviral transcriptional control pathways was no longer significant with adjustment for depressive symptoms. These findings suggest that depressive symptoms during pregnancy may influence infant immune function via inflammatory and antiviral transcriptional control pathways, with potential implications for subsequent health.

## 1. Introduction

The prenatal period is characterized by a rate of development unparalleled by any other period (Barker, 2007; Gluckman et al., 2005, 2010). Consequently, developing fetal neural and physiological systems are highly sensitive and responsive to environmental inputs. This heightened developmental plasticity helps guide neurobiological development in a manner that will promote survival and functioning according to environmental demands but can also present trade-offs for health and well-being (Bateson et al., 2014; Del Giudice et al., 2011; Nederhof & Schmidt, 2012). For example, fetal adaptations to a range of prenatal factors (e.g., maternal undernutrition, stress, cortisol, depression) can confer risk for mental and physical health conditions such as depression, ADHD, asthma, allergies, and cardiovascular disease, even extending through adulthood (Bush, 2024; Chen & Chen, 2021; Glover, 2011; Lautarescu et al., 2020; Monk et al., 2019; Rogers et al., 2020; Tung et al., 2024; Van den Bergh et al., 2017; Zijlmans et al., 2015). Such evidence underscores the importance of identifying the biological pathways through which prenatal factors shape offspring health outcomes. Although altered offspring immune system development is a putative pathway through which the prenatal environment influences offspring health and well-being, relatively few studies to date have tested the prospective associations of prenatal factors with offspring immune function in humans (Han et al., 2021a; Jiang et al., 2018; O’Connor et al., 2014; Veru et al., 2014; Zerbo et al., 2015). To address this research gap, the current study evaluates the associations of maternal depressive and anxiety symptoms during pregnancy with infant pro-inflammatory and antiviral gene expression at one month after birth.

Like many fetal neural and physiological systems, the development of the immune system unfolds through an ordered, carefully timed, and increasingly complex series of developmental events over the course of the prenatal period (Bateson et al., 2014; Gluckman et al., 2010; Han et al., 2021a; Howland et al., 2017; Veru et al., 2014). Several focal events occur in immune system development during the first trimester (e.g., formation of lymphocyte cell progenitors and secondary lymphoid organs), second trimester (e.g., expansion of T-cell populations), and third trimester (e.g., acquired immunocompetence and natural killer cell functionality), while the colonization of fetal immune cells occurs across the prenatal period (see Veru et al., 2014 for a review). Because of this rapid developmental sequence, the offspring immune system is highly sensitive to aspects of the in-utero environment. In rodents and non-human primates, experimentally inducing maternal stress during pregnancy leads to immune imbalances (e.g., enhanced Th2 relative to Th1 immune processes), enhanced pro-inflammatory cytokine production, and dampened stimulated immune responses in offspring (Marques et al., 2013; Veru et al., 2014). Although studies testing prospective associations of prenatal factors with immune processes in humans are less common, a few studies have linked perinatal risk factors to elevated pro-inflammatory cytokines and lower immunoglobin E (IgE) in offspring (Marques et al., 2013; Pedersen et al., 2018). Notably, the influence of prenatal factors on offspring immune function may be more potent than influences later in development (O’Connor et al., 2014), highlighting the importance of identifying the prenatal predictors of immune processes.

Further attention to the associations of maternal prenatal depressive and anxiety symptoms, in particular, with offspring immune processes is important for several reasons. First, elevated depressive and anxiety symptoms are a leading pregnancy complication, with estimates of clinically significant symptoms during pregnancy ranging up to 25 % for depression and 15 % for anxiety (Al-abri et al., 2023; Fairbrother et al., 2016; Furtado et al., 2018; Gavin et al., 2005). Second, prenatal depressive and anxiety symptoms have established implications for offspring developmental outcomes across domains (Meaney, 2018; Rogers et al., 2020). For example, one recent meta-analysis reported that prenatal symptoms of depression and anxiety were associated with offspring socio-emotional, language, motor, and cognitive outcomes extending across infancy, childhood, and adolescence (Rogers et al., 2020). Of note, several of the offspring outcomes linked with prenatal depressive and anxiety symptoms are immune-mediated (e.g., asthma, allergies, autism spectrum disorders) (Hameete et al., 2020; Han et al., 2021a; Han et al., 2021b; Knuesel et al., 2014; Meyer, 2019; O’Connor & Ciesla, 2022; Ronovsky et al., 2017). Accordingly, direct tests of associations of prenatal depressive and anxiety symptoms with offspring immune function may help to elucidate biological pathways through which maternal prenatal mood influences offspring health and well-being.

Several studies have linked prenatal depression and anxiety symptoms to offspring innate and adaptive immune system function. One of the first studies to test these associations in humans reported that infants of women who had anxiety during pregnancy showed reduced adaptive immunity following vaccination at six months (e.g., lower antibody responses) compared to infants of women who did not have anxiety during pregnancy (O’Connor et al., 2013). In another sample (*n* = 463), neonates of mothers who reported being ever depressed (either before or during pregnancy) showed lower stimulated IL-10 production in response to an allergen at birth, though there were no differences in stimulated TNF-α or IL-6 production (Hahn et al., 2019). Of note, one study found that the influences of prenatal depression on offspring immune function may extend through adulthood, reporting that maternal prenatal depression was associated with greater offspring C-reactive protein (CRP) levels at age 25 independently of other known risk factors for adult inflammation (e.g., childhood maltreatment, depression; *n* = 103) (Plant et al., 2016). However, other studies have reported null associations between prenatal depressive symptoms and offspring CRP in early adolescence (Maschke et al., 2021). Collectively, this research suggests that prenatal depression and anxiety may shape the development of the immune system, potentially contributing to elevated levels of circulating inflammation and dampened adaptive immunity.

A critical, yet understudied, way that prenatal maternal depressive and anxiety symptoms may influence offspring immune function is by regulating immune cell gene expression (Gillespie et al., 2019; Glover, 2011; Heim & Binder, 2012; Monk et al., 2019). Compared to measures of circulating inflammatory markers, immune cell gene expression reflects a distinct aspect of inflammatory biology (Lindsay et al., 2024; Young et al., 2020). Of note, however, immune processes, including pro-inflammatory protein production and antiviral immune activity, are primarily driven by the activation of signal transduction pathways that regulate the transcription (expression) of immune response genes (Irwin & Cole, 2011; Roy, 2019; Sloan & Cole, 2021; Smale, 2012). Moreover, immune cell signal transduction and gene expression are sensitive to social and environmental conditions like stress, adversity, and social isolation (Cole, 2013, 2019; Cole et al., 2012; Miller et al., 2009; Slavich et al., 2023; Slavich & Cole, 2013). For example, adversity-induced alterations to nervous system function can alter the regulation of pro-inflammatory and antiviral transcriptional pathways of immune cells, in a pathway known as the Conserved Transcriptional Response to Adversity (CTRA; Cole, 2019). In turn, CTRA transcriptome patterns hold implications for immune function and health, suggesting that altered pro-inflammatory and antiviral immune cell gene expression may be one potential pathway through which socioenvironmental conditions contribute to health risk (Cole, 2019; Slavich et al., 2023; Slavich & Cole, 2013). Although early socioenvironmental factors such as childhood adversity show meaningful associations with immune cell gene expression in children and young adults (Bower et al., 2020; Gillespie et al., 2019; Kuhlman et al., 2023, 2024; Méndez Leal et al., 2023), no studies to our knowledge have tested whether prenatal factors similarly relate to immune cell gene expression during infancy. Given that the developing fetus is highly responsive to the in-utero environment, it is plausible that prenatal depressive and anxiety symptoms could similarly influence offspring immune cell gene expression via fetal nervous system activity (Dipietro, 2012; DiPietro et al., 1996, 2008; DiPietro et al., 2018; Figueiredo et al., 2017; Pinto et al., 2023).

## 2. The current study

The aim of the current study was to test associations of maternal depressive and anxiety symptoms during pregnancy with pro-inflammatory and antiviral gene expression at in infants one month in a sample of 118 mother-infant pairs enrolled in a longitudinal study. We first conducted genome-wide transcriptional profiling of infant dried blood spots to map the empirical RNA correlates of maternal prenatal depressive and anxiety symptoms, considering both overall levels of symptoms and changes in symptoms throughout pregnancy. Next, we used bioinformatic analyses to test the hypothesis that these transcriptomic alterations might be attributed in part to elevated activity of the pro-inflammatory nuclear factor kappa-light-chain-enhancer of activated B cells (NF-κB) transcription control pathway and, as a secondary hypothesis, reduced activity of the interferon regulatory factor (IRF) antiviral transcription control pathway (consistent with the overall CTRA transcriptome pattern).

## 3. Methods

Participants in the present sample were mother-infant pairs enrolled in a longitudinal study, Healthy Babies Before Birth (HB3), designed to test the impact of antenatal maternal mood on birth outcomes and infant development. Data collection occurred from 2013 to 2018. Pregnant women were receiving prenatal care at prenatal clinics or private practices in Denver, Colorado and Los Angeles, California and were recruited into the study before completion of their 12th week of gestation. Inclusion criteria were 18 years of age or older with singleton intrauterine pregnancies. Exclusion criteria were current substance abuse diagnosis, HIV-positive status, current smoking, and multiple gestation. The study was comprised of three prenatal study visits that took place in early pregnancy (8–16 weeks gestation), mid pregnancy (20–26 weeks gestation), late pregnancy (30–36 weeks gestation), and three postpartum visits at 4–8 weeks postpartum, 5–7 months postpartum, and 11–13 months postpartum. Written informed consent was obtained from all participants who expressed interest and participants were compensated for each study visit. Each institution’s Institutional Review Board approved all protocols and procedures.

**Fig. 1.**
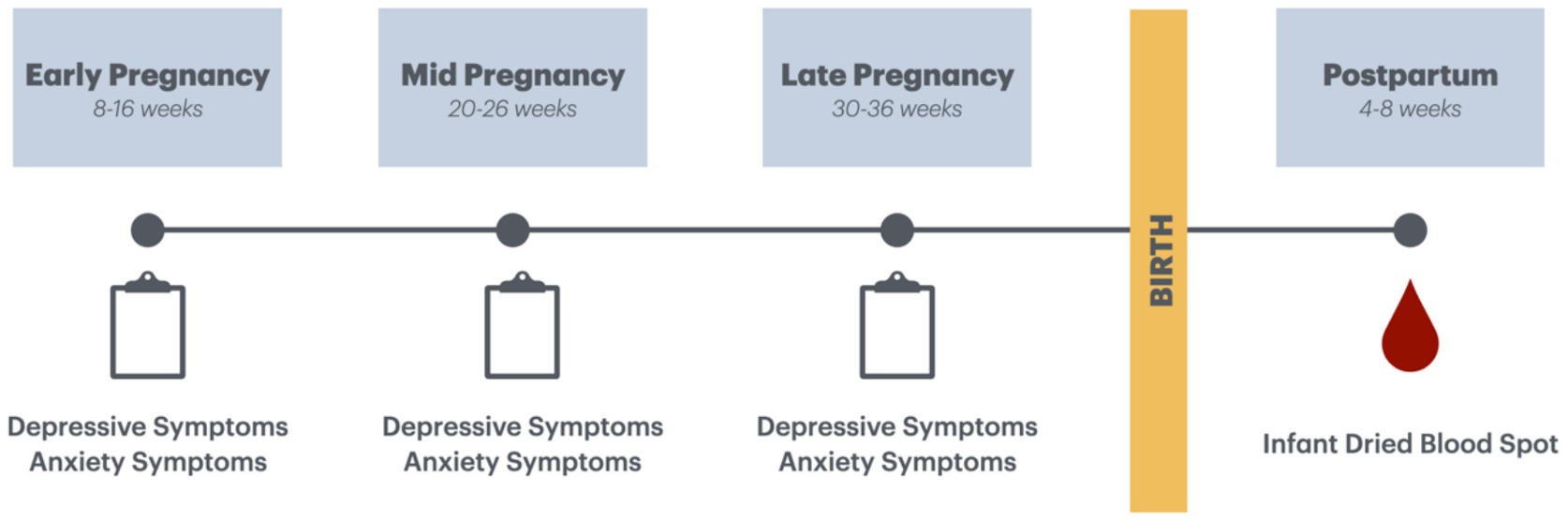
Overview of Study Design and Measures.

Of the 233 women enrolled in the study, the present sample includes participants who completed the study visits in early pregnancy, mid-pregnancy, late pregnancy, and at 4–8 weeks postpartum (*n* = 118). An overview of the study design is presented in Fig. 1 and complete sample characteristics are presented in Table 1. Mean maternal age at study entry was 31.9 years (*SD* = 6.17). Mean per capita annual household income adjusted for cost of living was $32,106 (*SD* = $28,698) and most of the sample had a bachelor’s (22.9 %) or graduate/professional degree (45.8 %). Nearly all participants were either married or in a relationship with the infant’s father at enrollment (96.7 %). Regarding racial and ethnic composition of the sample, most participants identified as White (87.0 %), and slightly less than one-third identified as Hispanic/Latina (30.9 %). Just over half of the infants were male (51.2 %). The sample was generally low risk medically concerning rates of gestational diabetes (2.5 %), hypertension (5.1 %), low birth weight (4.2 %), and preterm birth (4.2 %). About one-fifth of the sample had a C-section delivery (21.2 %).

### 3.1. Depressive and Anxiety Symptoms

We utilized the Patient Health Questionnaire (PHQ-9) to assess depressive symptoms at each prenatal study visit (Kroenke et al., 2001). The PHQ-9 is a multipurpose instrument for screening, diagnosing, monitoring, and measuring the severity of depression symptoms that is validated for use in pregnancy (Zhong et al., 2014; Zhong et al., 2015). Participants reported on the frequency of common depressive symptoms over the last 2 weeks on a scale of 0 (*not at all*) to 3 (*nearly every day*). Items were summed and total scores range from 0 to 27, with scores greater than or equal to 8 indicating clinically significant symptoms (Manea et al., 2012). The Overall Anxiety Severity and Impairment Scale (OASIS) was used to assess anxiety symptoms at each prenatal visit (Norman et al., 2006). The OASIS is a 5-item rating scale of anxiety frequency, intensity, behavioral avoidance, and functional impairment in the past week. Participants rated items on a scale of 0 (*low*) to 4 (*high*). Items were summed and total scores ranged from 0 to 20, with scores greater than or equal to 7 indicating clinically significant symptoms in non-clinical research samples (González-Robles et al., 2018). Cronbach’s alpha coefficients indicated acceptable reliability for the OASIS (α = 0.80–0.87) and PHQ-9 (α = 0.67–0.83) at each prenatal assessment in this sample.

Measures of depressive and anxiety symptoms over the course of pregnancy were operationalized with area under the curve measures to model patterns and cumulative levels of symptoms over the course of pregnancy (Pruessner et al., 2003). *Area under the curve with respect to ground* (AUCg) was calculated to model the overall levels of depressive and anxiety symptoms based on levels of symptoms at each prenatal study visit. Whereas AUCg reflects cumulative symptom levels, *area under the curve with respect to increase* (AUCi) was calculated to measure changes in symptoms over the course of pregnancy. AUCi is calculated based on changes in symptoms over time from baseline such that the sign and magnitude of AUCi represents the direction and the degree of change in symptoms across the three prenatal study visits. Each area under the curve measure reflects distinct information about repeated measurements (changes in symptoms [AUCi] vs. overall magnitude of symptoms [AUCg]), thus it is recommended to include both measures when analyzing data with repeated measures. Both measures were included in the present analyses based on evidence demonstrating that not only do levels of psychological distress fluctuate across pregnancy, but also that patterns of symptoms demonstrate distinct links with offspring outcomes as compared to overall symptom levels (Baron et al., 2017; Glynn et al., 2018; Irwin et al., 2020; Rinne et al., 2023; Santos et al., 2017).

### 3.2. Infant genome-wide transcriptional profiles

Infant genome-wide transcriptional profiles were obtained from a dried blood spot sample collected when infants were about one month of age. Trained research staff collected a non-fasting blood sample from infants on a dried blood spot card during a standardized heel stick blood draw. Heel stick blood draws are commonly used within this age range to elicit a mild stress response and assess neurobehavioral development (Davis et al., 2011; Gunnar et al., 2009). Dried blood spot cards were allowed to dry for 30 min and then stored at − 30 °C until assay. Dried blood spot samples were assayed by RNA sequencing as previously described (Ross et al., 2018, 2019). Briefly, RNA was extracted (Qiagen RNeasy) and converted to cDNA libraries using a high-efficiency mRNA targeted reverse transcription system (Lexogen QuantSeq 3′ FWD mRNA-Seq Library Prep Kit) and sequenced by Lexogen Services GmbH, using an Illumina NextSeq instrument, all following the manufacturers’ standard protocols for low-mass RNA samples. Assays targeted > 3 million single-stranded 100-nucleotide reads per sample (achieved mean = 3.1 million), each of which was mapped to the GRCh38 reference human transcriptome using the STAR aligner (average mapping rate = 89.0 %) and quantified as gene transcripts per million total mapped reads, with values floored at 1 to suppress spurious low-range variability and log2-transformed to reduce skew and heteroscedasticity for primary analyses (described below).

### 3.3. Statistical analysis

#### Primary Analyses.

Associations of maternal symptoms of depression and anxiety with infant pro-inflammatory gene regulation were assessed by TELiS bioinformatic analyses of NF-κB-binding motifs in the core promoter DNA sequences of genes that were up-regulated vs. down-regulated as a function of maternal depressive or anxiety symptoms, as previously described (Ross et al., 2018, 2019). In brief, linear statistical models were first applied to identify all gene transcripts that showed > 2-fold variation in average expression level across a 4-SD range of variation in maternal depressive symptoms or anxiety symptoms after statistically adjusting for covariates (see below for detail on covariates). Differentially expressed gene transcripts served as input into TELiS bioinformatics analyses (Cole et al., 2005) that quantified the relative prevalence of NF-κB transcription factor-binding motifs (identified by the TRANSFAC V$NFKAPPAB_01 position-specific weight matrix) in the core promoter DNA sequences of genes that were up- vs. down-regulated, with statistical testing based on bootstrap resampling of linear model residual vectors (which controls for within-individual correlation in gene expression). Secondary analyses used parallel methods to assess IRF transcription factor activity (V$IRF1_Q6).

#### Covariates.

Data on covariates were obtained via maternal interview or medical chart review. Analyses adjusted for infant covariates of length of gestation, biological sex, and age (days) at time of blood spot collection and maternal covariates of race and ethnicity, pre-pregnancy body mass index (BMI), infections during current or previous pregnancy (if applicable), and alcohol consumption during pregnancy. Infections during the current and previous pregnancy included a range of infections that could influence either maternal or offspring immune parameters (e.g., lower respiratory infections, bladder infections, bacterial infections, strep throat), coded dichotomously as any infections vs. none. Primary analyses adjusted for infant covariates only; secondary analyses controlled for maternal covariates as well as infant covariates to determine whether the pattern of results remained the same.

These covariates were selected for inclusion in analytic models based on associations with either maternal prenatal mood or offspring immune function in prior work or the present sample (e.g., Hahn et al., 2019; Marques et al., 2013; O’Connor et al., 2013; Plant et al., 2016). Breastfeeding and smoking during pregnancy were also considered as potential covariates given their role in influencing offspring immune parameters but were not included in final models due to low variability (*n* = 4 infants were not breastfeeding at the postpartum visit; *n* = 2 mothers reported smoking during pregnancy). In the current sample, maternal pre-pregnancy BMI was associated with overall depressive symptoms (*r* = 0.34, *p* < 0.001) and infections during pregnancy were associated with overall anxiety symptoms, *t*(110) = −2.48, *p* = 0.01). Length of gestation was associated with overall anxiety symptoms (*r* = −0.19, *p* = 0.04) and changes in anxiety symptoms (*r* = 0.38, *p* < 0.001). Associations of maternal mood measures with all infant and maternal covariates are presented in Supplementary Table 1.

#### Robustness Analyses.

Robustness analyses were conducted to determine whether prenatal depressive or anxiety symptoms were associated with patterns of infant immune cell gene expression independently of each other. Specifically, we tested whether associations of prenatal depressive symptoms with infant immune cell gene expression remained the same when prenatal anxiety symptoms were included in the model and, separately, tested whether associations of prenatal anxiety symptoms with infant immune cell gene expression remained the same when prenatal depressive symptoms were included in the model.

## 4. Results

### 4.1. Descriptive results

**Fig. 2.**
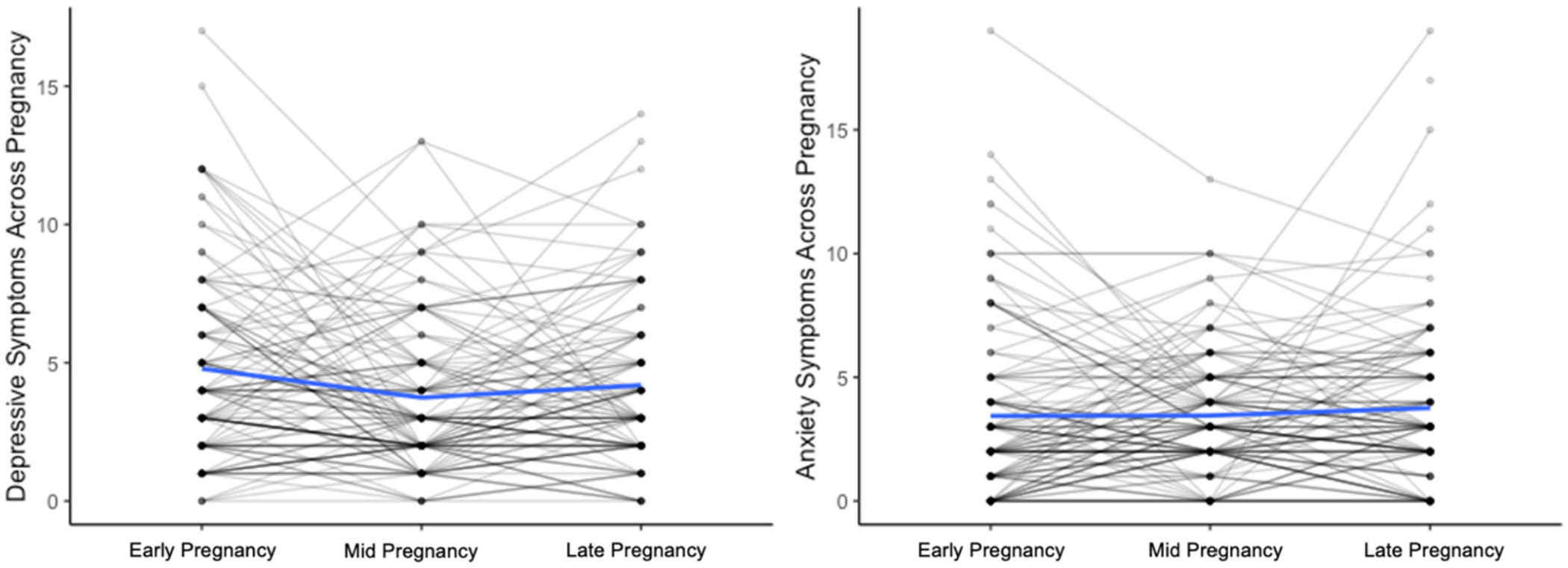
Individual trajectories of maternal depressive and anxiety symptoms from early pregnancy to late pregnancy. Mean levels of symptoms at each prenatal visit in the current sample are displayed in blue.

Descriptive statistics and bivariate correlations of prenatal depressive and anxiety symptoms are presented in the Table 2. Depressive symptoms were intercorrelated over trimesters of pregnancy (*r*’s = 0.34 to 0.52), as were anxiety symptoms (*r*’s = 0.46 to 0.52). As shown in Fig. 2, depressive and anxiety symptoms fluctuated over the course of pregnancy and the direction and degree of change varied across participants. On average, the sample reported low to moderate depressive and anxiety symptoms at each prenatal timepoint, with considerable variability in the sample. Rates of clinically significant depressive symptoms in the present sample were consistent with population estimates (20.3 % early pregnancy; 11.9 % mid pregnancy; 17.8 % late pregnancy), as were anxiety symptoms (17.8 % early pregnancy; 12.7 % mid pregnancy; 16.1 % late pregnancy) (Fairbrother et al., 2016; Furtado et al., 2018; Gavin et al., 2005).

### 4.2. Primary analysis results

**Fig. 3.**
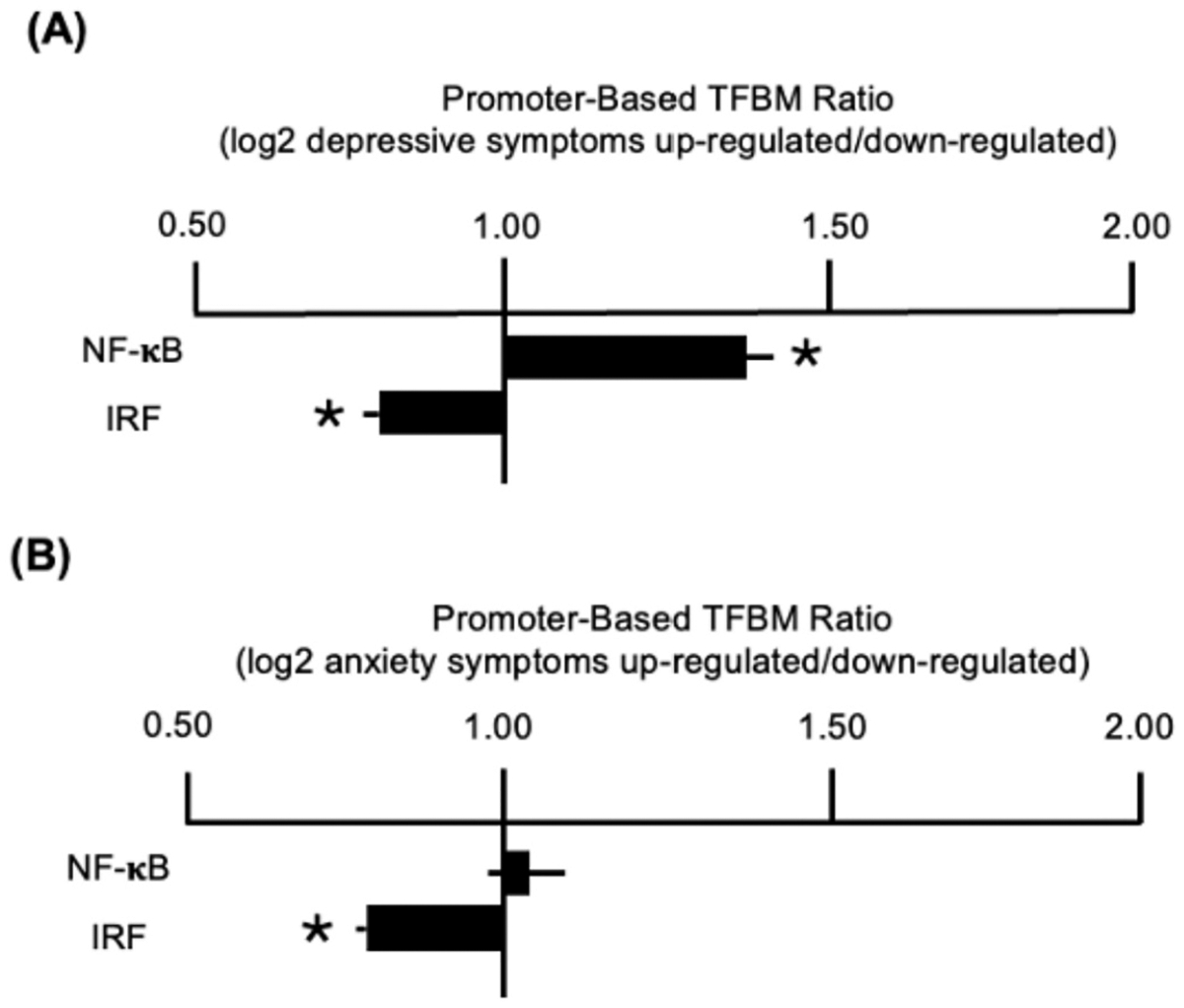
Promoter-based bioinformatics analyses of transcription factor binding motifs (TFBM) in up- vs. down-regulated genes associated with prenatal depressive (A) and anxiety symptoms (B).

Genome-wide analyses identified 428 gene transcripts that showed > 2.0-fold difference in average expression across a 4-SD range of prenatal depressive symptom levels (AUCg) after control for infant age, biological sex, and length of gestation (285 up-regulated and 143 down-regulated). In TELiS promoter-based bioinformatic analyses of those genes, results indicated significantly elevated levels of NF-κB activity (mean 1.48-fold ± SE 0.17 ratio of NF-κB response elements in promoter sequences of up-regulated vs. down-regulated genes, *p* = 0.012; Fig. 3A). Secondary analyses also linked maternal depressive symptom levels to reduced offspring IRF antiviral activity (0.73 ± 0.09, *p* < 0.001). Similar results emerged when analyses additionally controlled for maternal race/ethnicity, pre-pregnancy BMI, and infections and alcohol consumption during pregnancy (NF-κB: 1.47 ± 0.18, *p* = 0.024; IRF: 0.70 ± 0.08, *p* < 0.001). In robustness analyses that included maternal anxiety symptoms in analytic models, the pattern of associations with elevated levels of NF-κB activity and reduced IRF antiviral activity remained similar (NF-κB: 1.33 ± 0.14, p = 0.029; IRF: 0.86 ± 0.07, *p* = 0.038). Changes in depressive symptoms over the course of pregnancy (AUCi) were not significantly associated with pro-inflammatory or antiviral transcription factor activity.

In parallel analyses of the 748 gene transcripts that showed > 2.0-fold difference in average expression across a 4-SD range of prenatal anxiety symptom levels (571 up-regulated and 177 down-regulated), results showed no significant association with pro-inflammatory signaling (NF-κB: 1.06 ± 0.12, p = 0.640) but did indicate reduced IRF antiviral activity in models that controlled for infant and maternal covariates (IRF: 0.80 ± 0.06, p < 0.001; Fig. 3B). However, in robustness analyses that additionally controlled for depressive symptoms, prenatal anxiety symptoms no longer showed any significant associations with either pro-inflammatory or antiviral transcription factor activity (NF-κB: 0.92 ± 0.13, *p* = 0.507; IRF: 0.82 ± 0.27, *p* = 0.411). Changes in anxiety symptoms over the course of pregnancy (AUCi) were not significantly associated with proinflammatory or antiviral transcription factor activity in primary analyses. Estimates for all maternal mood predictors are presented in Table 3.

## 5. Discussion

The prenatal environment is theorized to shape offspring developmental trajectories primarily through adaptations to fetal biological systems, including the immune system (Howland et al., 2017; Monk et al., 2019). The present study tested associations of maternal depressive and anxiety symptoms during pregnancy with infant immune cell gene regulation at approximately one month of age. In primary study analyses, prenatal depressive symptom levels were associated with elevated NF-κB pro-inflammatory transcription factor activity. Secondary analyses of antiviral gene regulation indicated that depressive symptom levels were also associated with reduced Type I IRF transcription factor activity. These associations tracked the classical CTRA pattern of increased inflammatory activity accompanied by decreased Type I interferon activity and were independent of relevant maternal and infant covariates (e.g., prenatal infections, pre-pregnancy BMI, length of gestation, infant age, infant biological sex). Maternal anxiety symptom levels during pregnancy were associated with reduced IRF transcription factor activity, but not pro-inflammatory transcription factor activity, and were no longer related to antiviral transcription factor activity in robustness analyses that included depressive symptoms. Changes in depressive and anxiety symptoms over pregnancy were not associated with inflammatory or antiviral transcription factor activity in primary analyses. Overall, these findings provide novel evidence that depressive symptoms during pregnancy may shape pro-inflammatory and antiviral immune cell gene regulation in infancy.

Depressive symptoms during pregnancy are associated with a range of offspring mental and physical health outcomes, such as asthma, allergies, and neurodevelopmental disorders (Chen & Chen, 2021; Rogers et al., 2020; Tung et al., 2024). The developing fetal immune system is highly sensitive to prenatal inputs and several offspring health outcomes correlated with prenatal depressive symptoms are immune-mediated. However, few human studies have examined prospective associations of maternal prenatal depressive symptoms with offspring immune function. Here, levels of depressive symptoms over pregnancy were associated with infant gene expression, specifically up-regulated pro-inflammatory and down-regulated antiviral immune cell gene regulation. This pattern of findings is consistent with past research demonstrating that depressive symptoms during pregnancy may shape offspring immune processes, including stimulated immune responses in neonates and pro-inflammatory processes later in life (Hahn et al., 2019; Plant et al., 2016). Of note, the current study extends prior work by examining whether prenatal depressive symptoms are associated with the cellular activity of pro-inflammatory or antiviral transcriptional control pathways, compared to circulating inflammatory markers (e.g., CRP) or stimulated immune responses. This is important given that immune cell gene expression is particularly sensitive to early socioenvironmental conditions and contributes to many chronic disease processes such as cancer and cardiovascular disease (Bower et al., 2020; Gillespie et al., 2019; Heidt et al., 2014; Kuhlman et al., 2023, 2024; Méndez Leal et al., 2023; Sloan et al., 2010). Additionally, immune processes such as inflammation are primarily driven by immune cell gene expression (Cole, 2019). However, transcriptional control pathways are an aspect of inflammatory biology that is distinct from circulating pro-inflammatory markers; thus, there is variability in the strength of associations of inflammatory and antiviral gene expression with pro-inflammatory protein production (Heumann et al., 2025; Kuebler et al., 2015; Lindsay et al., 2024; Miklowitz et al., 2016; Sforzini et al., 2023; Young et al., 2020). Overall, the present findings provide novel evidence that prenatal depressive symptoms may influence offspring inflammatory biology at a transcriptional level, building upon past work that has focused on other measures of inflammatory biology (e.g., circulating pro-inflammatory cytokines).

In the present study, prenatal depressive symptoms predicted a pattern of gene regulation in infants consistent with the CTRA; specifically, up-regulated expression of pro-inflammatory genes and down-regulated expression of innate antiviral genes (Cole, 2019). The CTRA is known to be regulated by sympathetic nervous system (SNS) activation, with beta-adrenergic receptors transducing fight-or-flight neural signals to stimulate the activity of pro-inflammatory transcription factors such as NF-κB while simultaneously suppressing activity of the IRF family of transcription factors involved in Type I interferon signaling (Cole, 2019; Cole et al., 2012; Mondelli & Vernon, 2019; Roy, 2019; Smale, 2012). It is plausible that prenatal depressive symptoms operate through similar sympathetic nervous system-mediated pathways to influence this gene expression profile in infants. For example, prenatal depressive symptoms have been associated with other indicators of SNS activity including elevated fetal heart rate, greater heart rate reactivity to and slower heart rate recovery from stressors, and lower fetal heart rate variability (DiPietro, 2012; DiPietro et al., 1996, 2008; Kinsella & Monk, 2009). In turn, fetal SNS responses to prenatal maternal psychological distress may influence pro-inflammatory and antiviral transcription factor activity via beta-adrenergic signaling. Future tests of this hypothesized SNS-mediated pathway, as well as other maternal (e.g., inflammation; cortisol) and infant biological (e.g., gut microbiome) pathways, may help elucidate the biological mechanisms through which the prenatal environment influences offspring immune cell gene expression.

In contrast to depressive symptoms, anxiety symptoms during pregnancy were not related to pro-inflammatory immune cell gene expression in offspring. Although prenatal anxiety symptoms were associated with reduced antiviral immune cell gene expression when depressive symptoms were not in the model, this association did not persist in robustness analyses that included depressive symptoms. Together, these findings indicate differential patterns of associations of depressive symptoms with offspring immune function as compared to anxiety symptoms, and suggest that depressive symptoms show more consistent patterns of associations. There are several potential explanations for these results. First, past meta-analyses have similarly found that maternal depression during pregnancy is more strongly and more consistently associated with child socioemotional and health outcomes than is prenatal anxiety, including epigenetic profiles (Chen & Chen, 2021; Drzymalla et al., 2023; Madigan et al., 2018). This has been previously attributed to the links of depressive symptoms with physiological regulation and health behaviors (Dickens & Pawluski, 2018; Doom et al., 2024; Silva-Fernandes et al., 2024). Second, the present measure of depressive symptoms used in the present study assessed symptoms that resemble “sickness behaviors” (e.g., low energy, sleep disturbances, motor changes, changes in appetite, social withdrawal) and show particularly robust relationships with pro-inflammatory and antiviral processes in adults (Eisenberger et al., 2016; Irwin, 2019; Mac Giollabhui et al., 2020; Moriarity et al., 2020; Stewart et al., 2009). Conversely, the measure of anxiety symptoms focused on the severity of and impairment from fear and anxiety. Although anxiety during pregnancy has been associated with birth and offspring outcomes (Coussons-Read et al., 2012; Deer et al., 2024), it is plausible that anxiety is less directly implicated in the gene expression pathways studied here.

The present study was the first to our knowledge to examine how both levels of and changes in maternal depressive and anxiety symptoms across early, mid, and late pregnancy relate to infant immune cell gene expression. This is important given that there is substantial heterogeneity in symptom trajectories during pregnancy (e.g., Baron et al., 2017) and both subclinical symptom levels and changes in symptoms across pregnancy have been associated with offspring outcomes (Baron et al., 2017; Madigan et al., 2018; Rinne et al., 2022; Rogers et al., 2020). Although changes in depressive symptoms over the course of pregnancy have been previously linked with differences in infant biological regulation (e.g., cortisol reactivity; Laurent et al., 2011; Rinne et al., 2023), neither changes in depressive nor anxiety symptoms during pregnancy were associated with infant immune cell gene regulation. It could be that levels of depressive symptoms are differentially associated with fetal SNS activity compared to changes in depressive symptoms (Kinsella & Monk, 2009), with downstream implications for transcriptional control pathways (Cole, 2019; Cole et al., 2012; Mondelli & Vernon, 2019; Roy, 2019; Smale, 2012). Nonetheless, further research is needed to directly test this hypothesis. Future work should continue to examine symptom levels and changes for a more comprehensive understanding of how maternal prenatal mood shapes offspring development.

The present results were independent of relevant infant and maternal covariates, including sociodemographic and medical variables. Nonetheless, it may be informative in future research to also examine how sociodemographic and medical factors influence transcriptional control pathways in offspring. For example, although maternal depressive symptoms were associated with pro-inflammatory and antiviral transcriptional control pathways independently of length of gestation, future research could examine how length of gestation directly relates to these transcriptional pathways, particularly in samples with greater variability in length of gestation and higher frequency of preterm birth. Future research could also consider the role of delivery type and breastfeeding on infant pro-inflammatory and antiviral gene expression, particularly given that both have been associated with infant gut microbiome composition, which is implicated in immune system development (Donald & Finlay, 2023).

## 6. Strengths and Limitations

The present study has notable strengths. First, the collection of dried blood spots from infants at one month minimizes the potential for postnatal confounding influences on infant immune function. However, it is still plausible that early caregiving and postnatal stress could contribute to patterns of gene expression. Additionally, all assessments took place with trained research staff and validated procedures and measures at clinic or lab visits. The sample also included racially and ethnically diverse women and was heterogenous in both socioeconomic and geographic representation. Furthermore, whereas past studies have largely focused on how diagnoses of depression and anxiety during pregnancy relate to offspring immune function, the present study examined associations of prenatal mood with infant immune function based on assessments of symptoms during early, mid, and late pregnancy. This is a notable strength based on evidence that even subclinical variations in maternal depressive and anxiety symptoms during pregnancy influence offspring developmental trajectories (e.g., Rogers et al., 2020). Moreover, repeated assessments of symptoms allowed for comprehensive examination of how both prenatal symptom levels and changes shape offspring biological regulation.

The results of the present study should be considered in light of several limitations. It was beyond the scope of the present study to investigate the associations between these immune cell gene expression patterns and subsequent health or developmental outcomes or examine prenatal biological mechanisms explaining observed associations, which is an important area for future research. The present study also included one measure of infant immune function based on dried blood spots collected at one month of age. Given that the immune system continues to develop rapidly across infancy and childhood, repeated measures of immune processes may help to elucidate prenatal influences on trajectories of immune system development. Additionally, dried blood spots may confer a “noisier” transcriptome profile compared to blood samples obtained via venipuncture; however, collecting dried blood spots is minimally invasive and more feasible for obtaining measures of immune parameters in this age range (McDade et al., 2016). Similarly, dried blood spot RNA profiles are a measure of inflammatory biology that is distinct from and may not correlate with other measures of inflammatory biology; therefore, we cannot draw any conclusions about the associations of prenatal psychological distress with other immune processes in this sample (e.g., circulating pro-inflammatory cytokines; Lindsay et al., 2024; Young et al., 2020).

Most participants were enrolled in Los Angeles and primary analyses adjusted for sociodemographic factors that varied across sites (Somers et al., 2025), but factors that were not accounted for, such as altitude, could introduce differences across study sites. Rates of clinically elevated depressive and anxiety symptoms during pregnancy ranged from 12–20 %, consistent with population estimates (Fairbrother et al., 2016; Furtado et al., 2018; Gavin et al., 2005); however, this was not a clinical sample and less than 5 % of participants were taking medication for anxiety or depression during pregnancy. Thus, it is unclear whether these results would generalize to clinical samples or how medication use might influence infant immune function. Although meta-analyses indicate that antidepressant use during pregnancy is not associated with offspring neurodevelopment (e.g., Suarez et al., 2022), it may be important for future studies to examine how medication use might relate to maternal mood and offspring immune development.

A final limitation stems from the observational nature of this study, making it uncertain whether the observed differences in gene expression represent a causal effect of depressive symptoms on infant gene regulation or whether both factors are potentially regulated by a third variable (e.g., maternal somatic health). Future research with experimental designs, such as randomized clinical trials designed to reduce maternal depression and anxiety (e.g., the CARE Project; Davis et al., 2023, 2024), could help to clarify the causal pathways underlying the associations observed here.

## 7. Conclusions

Altered offspring immune system development is a putative pathway through which the prenatal environment shapes health outcomes, yet few studies test associations of prenatal factors with offspring immune function in humans. We found that maternal depressive symptom levels over the course of pregnancy were associated with elevated pro-inflammatory gene expression and decreased antiviral gene expression in infants at one month of age. By contrast, we observed no consistent associations of prenatal anxiety symptoms with infant immune cell gene expression, particularly when adjusting for maternal depressive symptoms during pregnancy. Overall, these findings advance scientific knowledge of prenatal influences on offspring immune function and provide novel evidence that prenatal depressive symptoms could shape child outcomes in part through alterations in immune cell gene regulation.

## Supplementary Material

Supplementary Material

## Figures and Tables

**Table 1:** Sample Characteristics (*n* = 118).

	M (SD) or % (n)
Maternal age at enrollment (years)	31.9 (6.2)
Infant age at postpartum visit (months)	1.3 (1.1)
Per capita income ($)	32,106 (28,698)
Breastfeeding at postpartum visit	96.6 % (4)
*Infant feeding > 50 % breastmilk*	77.1 %
Maternal educational attainment	
*High school or GED*	10.2 % (12)
*Some college*	18.6 % (22)
*College*	22.9 % (27)
*Graduate school*	45.8 % (54)
Relationship status with infant’s father	
*Married*	74.5 % (88)
*In a relationship*	21.2 % (25)
*Single*	1.7 % (2)
Maternal Race	
*White*	78.8 % (93)
*Black/African American*	9.3 % (11)
*Asian*	12.7 % (15)
Maternal Latina/Hispanic Ethnicity	27.1 % (32)
Study site	
*Los Angeles*	71.2 % (84)
*Denver*	26.2 % (31)
Primiparous	36.4 % (42)
Pre-pregnancy BMI	24.9 (6.0)
Gestational Age at Birth (weeks)	31.1 (1.6)
Birthweight (grams)	3,362 (612)
C-section delivery	21.2 % (25)
Infection during pregnancy	50 % (59)

Note. Per capita income was adjusted for cost of living at each study site.

**Table 2:** Descriptive statistics and bivariate correlations of prenatal depressive and anxiety symptoms.

	M	SD	Range	1	2	3	4	5	6
**1. Early Pregnancy Depressive Symptoms**	4.79	3.77	0–17	– -					
**2. Mid Pregnancy Depressive Symptoms**	3.74	2.93	0–13	0.34***	– -				
**3. Late Pregnancy Depressive Symptoms**	4.19	3.24	0–14	0.40***	0.50***	– -			
**4. Early Pregnancy Anxiety Symptoms**	3.44	3.65	0–19	0.52***	0.18	0.36***	– -		
**5. Mid Pregnancy Anxiety Symptoms**	3.45	2.75	0–13	0.08	0.25**	0.34***	0.50***	– -	
**6. Late Pregnancy Anxiety Symptoms**	3.76	3.46	0–19	0.17	0.05	0.45***	0.46***	0.46***	– -

*** *p* < 0.001;

** *p* < 0.01.

**Table 3:** Analysis Results.

	NF-κB			IRF		
	Est.	SE	*p*-value	Est.	SE	*p*-value
**Overall depressive symptom levels (AUC)**	1.33	0.14	0.029*	0.86	0.07	0.038*
**Changes in depressive symptoms (AUCi)**	1.06	0.14	0.674	0.92	0.07	0.198
**Overall anxiety symptom levels (AUC)**	0.92	0.13	0.507	0.92	0.06	0.208
**Changes in anxiety symptoms (AUCi)**	1.05	0.13	0.716	1.19	0.06	0.003**^a^

** *p* < 0.01;

** *p* < 0.05.

***Note***. Table presents model estimates from the fully adjusted, robustness analyses that controlled for covariates and included each maternal mood measure in the same model to determine whether associations in primary analyses were over and above the effect of other mood measures.

a Not significant in primary analyses.

## Data Availability

Data will be made available on request.

## Appendix A. Supplementary data

Supplementary data to this article can be found online at https://doi.org/10.1016/j.bbi.2025.106230.
