# Supplementary material for: Developmental origins of immune function: Maternal prenatal mood is associated with infant immune cell gene expression
Source: Brain Behav Immun. Author manuscript; Available in PMC 2026 Apr 30. (PMC13131241; doi:10.1016/j.bbi.2025.106230)
Supplement: Supplementary Material [file NIHMS2168429-supplement-Supplementary_Material.docx]

**Supplementary Materials**

| ***Supplementary Table 1.*** Associations of maternal mood measures with covariates | | | | |
| --- | --- | --- | --- | --- |
|  | **Overall Depressive Symptoms (AUC)** | **Changes in Depressive Symptoms (AUCi)** | **Overall Anxiety Symptoms (AUC)** | **Changes in Anxiety Symptoms (AUCi)** |
| **Infant Covariates** | **Test Statistic** | | | |
| Length of gestation | 0.02 | 0.01 | -0.19* | 0.38*** |
| Biological sex | -1.21 | 1.99* | 0.10 | 0.74 |
| Age | -0.17^ | 0.05 | -0.29** | -0.25** |
| **Maternal Covariates** | **Test Statistic** | | | |
| Race: White | 0.39 | 0.43 | 0.27 | -0.79 |
| Race: Black/African American | -0.74 | -1.07 | -0.07 | 0.59 |
| Race: Asian | 0.13 | -0.20 | -0.50 | 0.23 |
| Ethnicity: Latina/Hispanic | 0.78 | -0.06 | -1.04 | -0.35 |
| Pre-pregnancy BMI | 0.34*** | 0.13 | 0.08 | -0.18^ |
| Infections during pregnancy | -1.49 | 0.60 | -2.48 | 1.10 |
| Any alcohol consumption in pregnancy | 0.59 | 0.51 | -1.63 | -0.43 |
| *Note.* *** p < .001; ** p < .01; * p < .05; ^ p < .10. | | | | |
